# Urinary implications of retropubic dissection during abdominal wall reconstruction: Early results from a clinical quality assurance initiative

**DOI:** 10.1007/s10029-026-03771-y

**Published:** 2026-07-17

**Authors:** William C. Bennett, Emerson Lora, Mahamed O. Mohamed, Alvaro C. Carvalho, Noah X. Tocci, Erika M. Schmidt, Joseph Edwards, Ashley M. Mila-Hoff, Kimberly S. Miles, Sandip Vasavada, Clayton C. Petro, Ajita S. Prabhu

**Affiliations:** 1 Center for Abdominal Core Health, Department of General Surgery, Digestive Disease Institute, Cleveland Clinic Foundation, Cleveland, OH USA; 2 Center for Female Pelvic Medicine and Reconstructive Surgery, Glickman Urologic and Kidney Institute, Cleveland Clinic Foundation, Cleveland, OH USA; 3 Cleveland Clinic Lerner College of Medicine at the Case Western Reserve University School of Medicine, Cleveland, OH USA

**Keywords:** Retromuscular, TAR, Ventral hernia, Urinary incontinence, Quality assurance

## Abstract

**Purpose:**

Abdominal wall reconstruction with transversus abdominus release (TAR) requires space of Retzius dissection for retropubic mesh placement. Retzius space dissection has been associated with postoperative urinary incontinence (UI) in urologic literature, but implications of retropubic dissection are unreported for patients undergoing retromuscular ventral hernia repair. This quality assurance (QA) initiative sought to characterize pre- and postoperative UI prevalence at a high-volume hernia practice to inform risk–benefit discussions and surgical decision-making.

**Methods:**

Patients offered TAR, or who had previously undergone TAR, were given a Urinary Distress Inventory Short-Form (UDI-6) survey as a part of clinical workflow. UI was defined by UDI-6 score  ≥33.33, consistent with prior literature. A deidentified QA database captured responses and respondents’ age and gender. UI prevalence, UDI-6 total score, and patient characteristics were compared across preoperative, early postoperative, 1-year, and ≥ 2-year cohorts. A matched analysis was conducted for preoperative surveys that could be paired with a postoperative survey from the same patient. The study is reported according to the SQUIRE 2.0 guidelines.

**Results:**

Of 304 UDI-6 surveys, 82 (26.9%) met criteria for UI. Respondents were predominantly female (61.5%, n = 187) and median age was 62 years (IQR 53–69); 137 responses were collected preoperatively and 136 were collected in the early postoperative window. UI prevalence was higher at 37% (n = 49) within the preoperative cohort vs 18% with 6 months postoperatively (*P* = .0007). Thirty-three preoperative surveys were patient-matched to early postoperative surveys; among these, UI prevalence was again higher preoperatively at 30.3% (n = 10) vs 18.2% (n = 6, *P* = .003).

**Conclusion:**

Early results from this QA initiative provide reassurance against adverse urinary outcomes following retropubic dissection during TAR. Further prospective clinical trials may help to elucidate if there is a potential benefit to UI from TAR.

**Supplementary Information:**

The online version contains supplementary material available at 10.1007/s10029-026-03771-y.

## Introduction

Urinary incontinence (UI) is highly prevalent and can be exacerbated following abdomino-pelvic operations [1–3]. Retromuscular ventral hernia repair (VHR) with transversus abdominis release (TAR) frequently requires dissection through and mesh placement within the space of Retzius (referred to interchangeably as the retropubic space) for infraumbilical hernias and/or to ensure adequate inferior overlap [4]. Mesh-mediated pelvic inflammation and retropubic dissection have been associated with risk of UI in non-hernia postoperative courses [5–8], while other non-urologic pelvic procedures have been paradoxically associated with diminished UI [9]. UI is associated with significant direct costs on health systems and patient quality-of-life (QoL), however, to our knowledge, no existing literature considers UI or associated QoL after TAR [10–12]. Not only are pre- and postoperative UI prevalences unreported for patients requiring TAR, but our center’s population is predominantly female and aged > 60 years, suggesting a large proportion of patients are at an increased risk of UI at baseline [13]. To address this knowledge gap, we undertook a quality assurance (QA) initiative in a high-volume hernia practice. The Urinary Distress Inventory Short-Form (UDI-6) was incorporated into routine clinical care to characterize pre- and postoperative UI prevalence and potentially inform future risk–benefit counseling and surgical decision-making for patients undergoing complex abdominal wall reconstruction.

## Methods

### Study population

This prospective quality assurance (QA) initiative was conducted within a high-volume abdominal wall reconstruction practice at a quaternary care academic medical center starting in January 2025. While the QA initiative is ongoing, data within this report were collected through November 15, 2025. Per institutional practice for QA, data was handled in a deidentified fashion and patient participation, though optional, did not require written informed consent; institutional approval was obtained prior to analysis and external publication of findings. The QA initiative identified patients who presented to our outpatient surgical clinic and were offered ventral hernia repair via open TAR or had already undergone open TAR at our center. VHR with TAR was selected alone— rather than including other hernia repairs with entry into the Retzius space, such as Stoppa or Rives-Stoppa repairs— as it represents the most common procedure at our center and to simplify clinical workflow associated with the QA initiative. Notably, survey administration relied heavily on patient identification by clinical assistants, nurses, medical students, and residents with varying levels of anatomic and/or surgical knowledge; thus, simplification of patient identification by including only one procedure increased capture. Candidacy for TAR was the only inclusion criteria and presence of urostomy, altered urological anatomy (i.e. prostatectomy), or planned concurrent pelvic floor reconstruction were the only exclusion criteria for the preoperative cohort. Transurethral resection of prostate (TURP) procedures for benign prostatic hyperplasia were not excluded. Inclusion in a postoperative cohort required confirmation of Retzius space dissection via operative report review and the same exclusion criteria as the preoperative cohort were applied.

### Quality assurance initiative procedure

The primary aim of this QA initiative was to identify baseline prevalence of UI at various perioperative timepoints among patients who have undergone or may undergo TAR with retropubic dissection for large ventral hernias to assess for unrecognized urinary consequences of retropubic pelvic dissection and mesh placement. Secondary aims included characterization of UI severity in each cohort, comparison of UI burden between cohorts, and a sub-group analysis of female patients due to higher risk of UI. The results are reported according to the SQUIRE 2.0 guidelines. To accomplish this, eligible patients were offered a Urinary/Urogenital Distress Inventory Short-Form survey (UDI-6) at the conclusion of outpatient clinic visits [14]. The UDI-6 is a six-item questionnaire designed to assess the impact of UI and lower urinary symptoms of patient QoL by asking how much patients are bothered or distressed by urinary frequency, leakage, difficulty voiding, and lower abdominal/pelvic pain (range 0–100). The UDI-6 was chosen given its short length, characterization of several UI domains, validation in multiple populations, and focus on patient-perceived impact on life, as well as excellent sensitivity (97%) and specificity (84%) in distinguishing symptomatic vs asymptomatic UI in a binary fashion when a cut-off score of 33.33 is employed [15]. Therefore, we selected a score cut-off of ≥ 33.33% to define UI. UDI-6 subdomain scores were considered for irritative symptoms (items 1 and 2) and stress symptoms (items 3 and 4); subdomain scores were calculated via summation of scores and conversion to 100-point scales, as previously described [16].

Patients were informed of the QA effort prior to survey administration and participation was voluntary. Incomplete forms were discarded. Completed surveys were first recorded in the electronic medical record, then recorded in a deidentified dataset maintained for this project immediately thereafter. At time of data entry, medical records were referenced to assess for baseline urologic or pelvic disease for all patients to identify age, gender, and time (in days) after or before TAR; additionally, the operative and admission records of postoperative patients were reviewed for important contextual information. Operative notes were referenced to ensure space of Retzius dissection was conducted; at the same time mesh dimensions, mesh name, hernia dimensions, and operative duration were recorded. Perioperative admission records were referenced to document duration of indwelling urinary catheter (Foley catheter) utilization, if Foley utilization was non-pathway, and the reason(s) for prolonged Foley utilization. Our standard early recovery after surgery (ERAS) management of Foley catheters begins with placement of Foleys in all patients undergoing TAR following induction, then on POD1 or when the patient is ambulatory, whichever comes first. Reasons for deviations from ERAS Foley management were categorized as intensive care unit (ICU) monitoring, strict intake and output tracking (when concern for acute renal injury and/or hypovolemia), and urinary retention. The deidentified dataset was limited strictly to information required to conduct a sufficiently robust preliminary QA analysis. Time from surgery was utilized to assign survey responses to cohorts: preoperative (“Pre-Op”), early postoperative (“Early-Post”, range: 14–90 days), 1-year postoperative (“1-Year”, range: 180–545 days), and ≥ 2-years postoperative (“Distant”, range: ≥ 546 days). Notably, TAR did not need to occur within the QA project window, allowing characterization of UI at distant postoperative follow-up intervals.

Median age and percentage of patients-reporting female sex were also calculated for each cohort. Demographic data were assessed for similarity across survey cohorts and historical values for our practice. Sensitivity analyses considering baseline urologic/pelvic disease, Foley utilization, and mesh characteristics were performed to increase interpretability of findings.

If preoperative responses were present in a medical record when postoperative responses were being recorded, the preoperative responses were identified in the dataset, and a dummy identifier was used to link the pre- and postoperative responses. The dummy identifiers were non-consecutive two-character alphanumeric values (i.e. a1, b1, a2…) which were not recorded in the medical record, held no relation to the patient, and cannot be used to identify patients.

### Characterization of concurrent and historical populations

To assess the representativeness of survey respondent cohorts with historical cohorts, Epic SlicerDicer was used to extract summary statistics regarding age and gender of patients undergoing open TAR at our institution. A SlicerDicer module containing all surgeries and invasive procedures was used as the base population, then filtered for surgeries conducted by participant surgeons. A second filter was applied to capture all open ventral hernia repairs with defect size greater than 10 cm. Mean, standard deviation, median and interquartile values were captured for age, and percentages were captured for gender across two time periods. The first time period (“concurrent”) matched the data collection period (January 1, 2025 – November 15, 2025) to assess for asymmetry between patients who received surveys and all open TAR patients during the QA initiative. The second time period (“historical”) covered two calendar years prior to the QA initiative (January 1, 2023 – December 31, 2024) to assess for asymmetry between patient populations during the QA initiative and historical patient populations.

### Statistical analysis

Descriptive statistics were utilized to produce median and interquartile range (IQR) for age and UDI-6 scores within each cohort. Mann–Whitney U tests were utilized for comparison of independent continuous variables, Wilcoxon matched signed-rank test was utilized for the patient-matched analysis, and Spearman rank correlation for continuous variable associations. Fisher’s exact test was used for all categorical comparisons, except for the patient-matched analysis which utilized McNemar’s Chi-Square. Unpaired t test was utilized to compare cohort demographics vs historical values via mean, standard deviation, and cohort size. Primary comparisons were repeated featuring cohorts restricted to patients without baseline urologic or pelvic pathology and excluding surveys captured within 21 and 30 days of surgery as sensitivity analyses to assess robustness to confounding. Statistical significance was predetermined at *P* ≤ 0.05. All analyses were performed with GraphPad Prism version 10.4.1 for macOS (GraphPad Software; Boston, MA; www.graphpad.com) or R version 4.4.1 (R Core Team, 2024)[17] using RStudio version 2026.01.0 (Posit team, 2026)[18] with dplyr [19], readxl [20], and writexl packages [21].

## Results

A total of 301 eligible surveys were collected. Median respondent age was 62 years (IQR 53–69), and respondents were predominantly female (62.8%, n = 189). The Pre-Op cohort had 138 responses, the Early-Post cohort had 133 responses, the 1-Year cohort had 23 responses, and the Distant cohort had just 7.

Demographics were comparable across cohorts (Table 1). Age was similar between Pre-Op (61 years, (IQR 52–69)) and Early-Post (63 years, [IQR 54–69]), 1-Year (60 years, [IQR 58–63.5]), and Distant (54 years, (IQR 48–58.5)) cohorts (*P* > 0.2 across all); age distribution was similar among study cohorts and historical populations (Supplemental Results). Females comprised 60.9% (n = 84) of Pre, 64.7% (n = 86) of Early-Post, 65.2% (n = 15) of 1-Year, 57.1% (n = 4) of the Distant cohort, which were statistically similar; female prevalence was similar between historical control populations and all study cohorts (Supplemental Results).

**Table 1:** Demographic, Urologic/Pelvic medical history, perioperative indwelling catheterization, and hernia repair characteristics

Variable	All^a^	Pre-Op	Early-Post	*P*^*b*^	1-Year	*P*^*c*^	Distant	*P*^*d*^
n	301	138	133		23		7	
Age (y), Median (IQR)	62.0 (53.0–69.0)	60.5 (52.0–69.0)	63.0 (54.0–69.0)	0.212	60.0 (58.0–63.5)	0.892	54.0 (48.0–58.5)	0.247
Female, n (%)	189 (62.8%)	84 (60.9%)	86 (64.7%)	0.532	15 (65.2%)	0.818	4 (57.1%)	1.000
Time since TAR (d), Median (IQR)	—	—	25 (18–32)	N/A	381 (286–441)	N/A	1964 (1570–2666)	N/A
Baseline Urologic/Pelvic Disease								
Any current, n (%)	56 (18.6%)	25 (18.1%)	24 (18.0%)	1.000	6 (26.1%)	0.394	1 (14.3%)	1.000
BPH (males), n (%)	41 (36.6%)	17 (31.5%)	18 (38.3%)	0.533	6 (75.0%)	0.043	0 (0.0%)	0.547
Cystocele/POP (females), n (%)	10 (5.3%)	4 (4.8%)	5 (5.8%)	1.000	0 (0.0%)	1.000	1 (25.0%)	0.212
Foley Catheter Utilization, n (%)	163 (100%)	—	133 (100%)	N/A	23 (100%)	N/A	7 (100%)	N/A
Foley use off ERAS pathway, n (%)	33 (20.2%)	—	24 (18.0%)	N/A	6 (26.1%)	N/A	3 (42.9%)	N/A
Foley time (h), Median (IQR)	25.4 (22.6–45.8)	—	25.3 (22.5–43.0)	N/A	25.2 (22.7–50.2)	N/A	46.0 (41.8–68.8)	N/A
Second Foley placed, n (%)	11 (6.7%)	—	8 (6.0%)	N/A	3 (13.0%)	N/A	0 (0.0%)	N/A
Mesh Characteristics								
Polypropylene (PP), n (%)	151 (92.6%)	—	123 (92.5%)	N/A	23 (100.0%)	N/A	5 (71.4%)	N/A
Weight ≥ 75 g/m^2^, n (% of PP)	101 (66.9%)	—	86 (69.9%)	N/A	14 (60.9%)	N/A	1 (20.0%)	N/A
Mesh area (cm^2^), Median (IQR)	900.0 (900.0–1600.0)	—	900.0 (900.0–1200.0)	N/A	900.0 (900.0–1600.0)	N/A	900.0 (900.0–2262.5)	N/A
Hernia area (cm^2^), Median (IQR)	322.0 (210.0–432.0)	—	313.5 (200.0–418.5)	N/A	315.0 (222.8–525.0)	N/A	405.0 (332.5–451.0)	N/A
Mesh-to-hernia area ratio, Median (IQR)	3.6 (2.6–4.8)	—	3.6 (2.6–5.0)	N/A	3.7 (2.9–4.2)	N/A	4.0 (2.4–4.8)	N/A

Abbreviations: IQR, interquartile range; BPH, benign prostatic hyperplasia; POP, pelvic organ prolapse; ERAS, early recovery after surgery; PP, Polypropylene

Mann–Whitney U test utilized for continuous variable comparisons and Fisher’s Exact test used for categorical variable comparisons

a. Considers only postoperative cohorts for rows regarding catheterization, mesh characteristics, and hernia size

b. Early-Post vs Pre-Op

c. 1-Year vs Pre-Op

d. Distant vs Pre-Op

### Symptomatic UI prevalence and UDI-6 scores

Overall prevalence of symptomatic UI was 26.6% (n = 80). Pre-Op UI prevalence was 38.4% (n = 53) and higher than the Early-Post (18.0%, n = 24; *P* < 0.001) and 1-Year (8.7%, n = 2; *P* = 0.004) cohorts. The Distant cohort symptomatic UI prevalence (14.3%, n = 1; *P* = 0.258) was not statistically different from Pre-Op.

Median overall UDI-6 score was 16.7 (IQR 5.6–33.3). Median UDI-6 score among the Pre-Op cohort was 22.2 (IQR 11.1–44.4) which was significantly higher than the Early-Post cohort median of 11.1 (IQR 0.0–27.8; *P* < 0.001) and the 1-Year cohort (median 11.1, (IQR 5.6–27.8); *P* = 0.023). The Distant cohort UDI-6 scores (median 11.1, (IQR 2.8–16.7)) were not statistically different from the Pre-Op cohort (*P* = 0.07).

Irritative UI sub-domain scores for the Pre-Op cohort (median 33.3, (IQR16.7–50.0)) were statistically higher than Early-Post (median 16.7, (IQR 0.0–50.0); *P* = 0.014) and Distant (median 0.0, (IQR 0.0–16.7); *P* = 0.035) scores, but not the 1-Year cohort. A statistically significant difference in stress UI scores was identified between every postoperative cohort vs the Pre-Op cohort (Table 2).

**Table 2:** Aggregate characterization of urinary incontinence at specified pre- and post-operative intervals

Variable	All	Pre-Op	Early-Post	*P*^*a*^	1-Year	*P*^*b*^	Distant	*P*^*c*^
n	301	138	133		23		7	
Urinary incontinence, n (%)	80 (26.6%)	53 (38.4%)	24 (18.0%)	< 0.001	2 (8.7%)	0.004	1 (14.3%)	0.258
UDI-6 Score, Median (IQR)	16.7 (5.6–33.3)	22.2 (11.1–44.4)	11.1 (0.0–27.8)	< 0.001	11.1 (5.6–27.8)	0.023	11.1 (2.8–16.7)	0.070
Irritative Score, Median (IQR)	16.7 (0.0–50.0)	33.3 (16.7–50.0)	16.7 (0.0–50.0)	0.014	16.7 (0.0–41.7)	0.065	0.0 (0.0–16.7)	0.035
Stress Score, Median (IQR)	0.0 (0.0–33.3)	16.7 (0.0–33.3)	0.0 (0.0–16.7)	< 0.001	0.0 (0.0–16.7)	0.014	0.0 (0.0–0.0)	0.041

Abbreviations: IQR, interquartile range

Mann–Whitney U test utilized for continuous variable comparisons and Fisher’s Exact test used for categorical variable comparisons

a. Early-Post vs Pre-Op

b. 1-Year vs Pre-Op

c. Distant vs Pre-Op

### Female patient analysis

Similar trends were observed when male respondents were removed and only females were considered; ages were similar across female-only respondent cohorts (Table 3). Overall UI prevalence mildly increased to 29.6% (n = 56). Prevalence of UI was again lower for the Early-Post (23.3%, n = 20; *P* = 0.014) and 1-Year (6.7%, n = 1; *P* = 0.009) cohorts vs the Pre-Op cohort (40.2%, n = 33); no UI was observed in the Distant cohort which featured only 4 female patients. UDI-6 scores were higher in the Pre-Op cohort (median 27.8, [IQR 12.8–44.4]) vs the Early-Post (median 16.7, (IQR 5.6–27.8); *P* < 0.001), 1-Year (median 16.7, (IQR 5.6–27.8); *P* = 0.021), and Distant (median 8.3, (IQR4.2–13.9); *P* = 0.038) cohorts. Significantly lower scores in both irritative (P = 0.032) and stress (P < 0.001) subdomain scores were observed in the female Early-Post cohort compared to female Pre-Op respondents. Lower stress scores were also observed in the female 1-Year cohort (P = 0.008) (Table 3).

**Table 3:** Demographic and reported urinary incontinence outcomes among female patients

Variable	All	Pre-Op	Early-Post	*P*^a^	1-Year	*P*^b^	Distant	*P*^*c*^
n	189	84	86		15		4	
Age (y), Median (IQR)	61.0 (53.0–69.0)	61.0 (53.0–69.2)	62.5 (54.0–69.0)	0.984	61.0 (56.0–65.5)	0.895	52.0 (49.0–59.0)	0.366
Female, n (%)	189 (100.0%)	84 (100.0%)	86 (100.0%)	N/A	15 (100.0%)	N/A	4 (100.0%)	N/A
Time since TAR (d), Median (IQR)	—	—	24 (17–32)	N/A	379 (276–434)	N/A	1570 (1488–1903)	N/A
Urinary incontinence, n (%)	56 (29.6%)	35 (41.7%)	20 (23.3%)	0.014	1 (6.7%)	0.009	0 (0.0%)	0.148
UDI-6 Score, Median (IQR)	22.2 (5.6–33.3)	27.8 (12.8–44.4)	16.7 (5.6–27.8)	< 0.001	16.7 (5.6–27.8)	0.021	8.3 (4.2–13.9)	0.038
Irritative Score, Median (IQR)	33.3 (0.0–50.0)	33.3 (16.7–54.2)	25.0 (0.0–50.0)	0.032	16.7 (0.0–50.0)	0.109	0.0 (0.0–4.2)	0.014
Stress Score, Median (IQR)	16.7 (0.0–33.3)	33.3 (0.0–37.5)	0.0 (0.0–33.3)	< 0.001	0.0 (0.0–16.7)	0.008	0.0 (0.0–8.3)	0.108

Abbreviations: IQR, interquartile range; TAR, transversus abdominis release

Mann–Whitney U test utilized for continuous variable comparisons and Fisher’s Exact test used for categorical variable comparisons

a. Early-Post vs Pre-Op

b. 1-Year vs Pre-Op

c. Distant vs Pre-Op

### Matched analysis

Of the 138 preoperative surveys, 33 (24%) could be matched to a postoperative survey by the same patient. Median age of matched patients was 65 years (IQR 51–69) and 63.6% were female (n = 21) (Table 4). Median time from surgery was 26 days (IQR 21–29) in the postoperative group. The preoperative symptomatic UI prevalence among matched surveys (33.3%, n = 11) was numerically higher than the postoperative rate of 18.2% (n = 6), though this difference did not reach statistical significance in the McNemar's paired analysis (*P* = 0.059). Group UDI-6 scores were also higher in the preoperative group (median 22.2, (IQR 11.1–38.9)) compared to the postoperative group (median 11.1, (IQR 0.0–27.8); *P* = 0.004). The matched analysis identified a significant reduction in stress symptom scores postoperatively (Pre-Op median 16.7 vs Early-Post 0.0; P = 0.044). Irritative scores were numerically lower in the Early-Post group, but did not reach significance (P = 0.052).

**Table 4:** Demographic and reported urinary incontinence outcomes, matched cohort

Variable	Pre-Op	Early-Post	*P*
n	33	33	
Age (y), Median (IQR)	65.0 (51.0–69.0)	65.0 (51.0–69.0)	N/A
Female, n (%)	21 (63.6%)	21 (63.6%)	N/A
Time since TAR (d), Median (IQR)	—	26 (21–29)	N/A
Urinary incontinence, n (%)	11 (33.3%)	6 (18.2%)	0.059
UDI-6 Score, Median (IQR)	22.2 (11.1–38.9)	11.1 (0.0–27.8)	0.004
Irritative Score, Median (IQR)	33.3 (16.7–50.0)	16.7 (0.0–50.0)	0.052
Stress Score, Median (IQR)	16.7 (0.0–33.3)	0.0 (0.0–33.3)	0.044

Abbreviations: IQR, interquartile range; TAR, transversus abdominis release

Wilcoxon signed rank test utilized for continuous variable comparisons and McNemar’s chi-square test used for paired categorical variable comparisons

In a follow-up analysis of cohort comparability between matched and unmatched participants conducted to assess possible selection bias within matched cohort, no differences were observed between the matched and unmatched participants among the Pre-Op or Early-Post cohorts regarding age, gender, UI prevalence, UDI-6 score, UDI-6 irritative sub-score, UDI-6 stress sub-score, presence of urologic/pelvic floor pathology (any), BPH prevalence, or cystocele/POP prevalence (Supplement, eTable 1).

### Perioperative urinary catheter utilization

Perioperative catheter placement after induction and before incision time was confirmed in all postoperative patients (n = 162, 100%), consistent with our standard practice. Median duration of catheterization was 25.4 h (IQR 22.5–42.8) (Supplement, eTable 2). Thirty-three (20%) required indwelling catheterization beyond the standard ERAS pathway for one or more of the following reasons: strict intake and output monitoring (16.6%), per ICU protocol (8.0%), acute urinary retention (1.8%), or epidural therapy (0.6%). No patients were discharged with an indwelling catheter or required a third catheterization.

In sensitivity analyses considering Foley utilization and postoperative UI outcomes within the Early-Post cohort, no differences between standard ERAS pathway Foley utilization and non-pathway Foley utilization were identified regarding UI prevalence (17.4% vs 20.8%, respectively; *P* = 0.770) or UDI-6 score (median score 11.1, (IQR 0.0–27.8) vs 13.9, (IQR 5.6–27.8), respectively; *P* = 0.556) (Supplement, eTable 3 A). No differences in UI prevalence (*P* = 0.635) or UDI-6 score (*P* = 0.328) were observed between those who did or did not require a second Foley catheterization (Supplement, eTable 3B). Within the matched cohort, no correlation was identified between magnitude of total UDI-6 score change or duration of catheterization (rho = −0.181, *P* = 0.312), nor for the irritative or stress subdomain scores (Supplement, eTable 3C).

### Baseline urologic and pelvic floor pathology

Overall prevalence of any pathology was 18.6% (n = 56), which was predominantly related to males with BPH (n = 41, 36.6%), and rates of baseline pathology were comparable across all cohorts, other than a higher prevalence of males with BPH in the 1-Year cohort (n = 6, 75.0%) vs the Pre-Op cohort (n = 17, 31.5%; *P* = 0.043) (Table 1). An association analysis demonstrated no significant association between presence of baseline pathology and UI prevalence or UDI-6 score in the preoperative cohort (Supplement, eTable 4A). A subsequent sensitivity analysis considering only the 113 Pre-Op and 109 Early-Post patients without baseline pathology redemonstrated results of the primary analysis: lower UI prevalence (37.2% vs 18.3%, respectively; *P* = 0.003) and lower UDI-6 scores (median 22., (IQR 5.6–44.4) vs 11.1, (IQR 0.0–27.8), respectively; *P* = 0.001) favoring the Early-Post cohort (Supplement, eTable 4B).

### Operative and mesh characteristics

Retzius space dissection was confirmed in all cases (n = 163, 100%). Median hernia width was 15.0 cm (IQR 11.0–19.0), 6 (3.7%) hernias included a flank component, and 9 (5.5%) hernias featured a parastomal component. Most repairs employed uncoated polypropylene (n = 151, 92.6%); two-thirds (n = 101) of polypropylene meshes were heavy-weight (HWPP, ≥ 75 gm/m^2^) and the remaining 50 (33.1%) were medium-weight (MWPP). Six repairs used coated polytetrafluoroethylene (PTFE; biosynthetic permanent), 4 used poly(L-lactide-co-trimethylene carbonate) (PLLA/TMC; synthetic absorbable), and 2 used polyester (permanent synthetic).

An analysis of mesh weight featuring only HWPP vs MWPP to control for material identified a possible non-significant signal toward reduced UI prevalence in HWPP (n = 11, 12.8%) vs MWPP (n = 10, 27.0%; *P* = 0.069) and a significant reduction in UDI-6 stress score (median 0.0, (IQR0.0–16.7) vs 0.0, (IQR0.0–33.3); *P* = 0.02) (Supplement, eTable 5A). No differences in UDI-6 total score (*P* = 0.139) or irritative score (*P* = 0.145) were observed, but hernia and mesh size were significantly greater in the MWPP cohort (eTable 5A). Therefore an association analysis was conducted considering total mesh size, hernia size, and mesh:hernia size ratio vs change in UDI-6 scores among matched Pre-Op and Early-Post patients. An inverse association between hernia size and change in UDI-6 score (rho = −0.361; *P* = 0.039) was the only significant relationship observed between size variables and UDI-6 score (Supplement, eTable 5B). UDI-6 and mesh material associations could not be similarly evaluated given prohibitively low non-polypropylene cohort sizes.

### Time-dependent variation in early postoperative UI

In sensitivity analyses conducted to evaluate possible confounding of a broad survey window (14–90 days) or time-dependent physiologic changes following mesh-based ventral hernia repair within the Early-Post cohort, no significant correlation was observed between time since surgery and UDI-6 score (Spearman rho = 0.081, P = 0.353), nor between days post-TAR and within-patient UDI-6 score change in the matched cohort (rho = − 0.071, P = 0.694)— indicating UDI-6 scores did not vary as a function of survey timing within the observation window (Supplement, eTable 6A). To specifically address the concern that pain-related activity restriction or postoperative inflammation in the most acute recovery period may have artifactually suppressed UDI-6 scores, we repeated the primary Pre-Op vs Early-Post comparison excluding patients surveyed within 21 days (n = 88) and 30 days (n = 43) of surgery. In both restricted analyses, the decreased UI prevalence (> 21 days: *P* < 0.001; > 30 days: *P* < 0.001) and UDI-6 scores (> 21 days: *P* = 0.001; > 30 days: *P* = 0.001) for Early-Post patients relative to the Pre-Op cohort remained statistically significant, as did differences in irritative and stress UI sub-domain scores (Supplement, eTable 6B). Then, a final sub-window analysis to test replicability of results across the broad 14–90 day Early-Post window demonstrated UDI-6 scores were significantly lower than preoperative values at 14–30 days (P = 0.011), 31–60 days (P = 0.007), and 61–90 days (P = 0.029) post-TAR (Supplement, eTable 6C).

## Discussion

These initial results from an ongoing QA initiative demonstrate no apparent increase in UI— neither prevalence or patient-reported symptom burden— following complex abdominal wall reconstruction with TAR with Retzius space dissection for multiple postoperative populations when compared against a preoperative population. The findings are reassuring given urologic literature suggesting space of Retzius dissection is associated with increased postoperative UI while no prior investigations have assessed for adverse urinary outcomes following Retzius space dissection during hernia repair [7, 13]. The QA initiative was successful in collecting over 300 responses from a representative sample of the center’s patient population, as demographic data (sex and age) among survey respondents demonstrated homogeneity with historic populations, and well-characterized UI for preoperative and early postoperative populations.

That nearly 60% of open TAR patients were female and median age was 62 years (IQR 53–69), substantiates anecdotal concerns regarding our patient populations’ baseline risk of UI based on age and sex. The identification of a 41% UI prevalence among preoperative female respondents is slightly higher but relatively consistent with the estimated prevalence of 32.4 to 38% among all US women by the National Health and Nutrition Examination Survey (NHANES) [13, 22]. Likewise we demonstrated a 33% prevalence among preoperative female respondents aged ≥ 60 years which agrees with the NHANES estimate of 33% to 41% UI prevalence for the same population [13, 22].

The absence of adverse UI outcomes following TAR provides reassurance against fears of induced/exacerbated UI secondary to space of Retzius dissection seen in urologic literature. Multiple factors can account for this discrepancy with urologic literature. Foremost, TAR and traditional prostatectomy are fundamentally different operations, sharing only dissection of the retropubic space. Regardless of the Retzius-sparing vs conventional approach, pelvic and urethral anatomy are substantially altered and postoperative UI is inherent to the procedure regardless of approach; demonstrated by the observation that Retzius-sparing prostatectomy does not avoid postoperative UI altogether [23–25]. The same is not necessarily true of ventral hernia repair via TAR in which pelvic anatomy and the genitourinary system are largely undisturbed (other than dissection into the retropubic space, the extent of which is also limited relative to prostatectomy). Further the mechanism of UI seen after prostatectomy likely differs from the UI experienced by our population. Approximately 67 to 92% of postoperative UI after prostatectomy is intrinsic sphincter deficiency (ISD), a cause of stress incontinence [26]. Not only does our predominantly female population differ from the male population of urologic literature, but so does the predominant mechanism of UI in women. While stress UI is also the most common type of UI, accounting for 37% of UI in women, it largely relates to pelvic floor dysfunction— sometimes with overlapping ISD, but rarely ISD alone [13, 27]. Further, urgency UI and mixed UI account for 22% and 31.3% of UI in the general female population, respectively, which are entirely unrelated to the ISD stress incontinence observed after prostatectomy [13]. Therefore, the absence of adverse urinary outcomes following TAR (vs traditional prostatectomy) may simply result from the substantial differences in operative course and patient population between the procedures.

Limitations in methodology may be responsible for the apparent improvement in UI after TAR and these are discussed below; however, we can speculate as to why TAR with Retzius dissection might confer improved urinary function in a population with stress or mixed UI despite violation of the retropubic space. First, repair of a large hernia defect may provide a mechanistic benefit which ameliorates UI. Large defects compromise the structural integrity of the abdominal wall and likely disrupt coordinated pressure dynamics maintained between the pelvic floor, abdominal wall, and diaphragm. The consequences of this have been previously suggested in literature considering postpartum women with severe rectus diastasis where increasing width was associated with UI— though diastases is, of course, a distinct disease process from ventral hernias [28, 29]. A recent study of patients with stress UI who underwent abdominoplasty with diastasis repair demonstrated significant improvement in UI which suggests restoration of ventral abdominal wall integrity could improve urinary continence [30]. Our results also suggest the possibility of this mechanism, as the decrease in UDI-6 score among matched patients was greater among larger hernias. Alternatively, differences in operative manipulation within the Retzius space may suggest the contrasting urinary outcomes between prostatectomy literature and our findings. Evidence suggests Retzius-sparing prostatectomy preserves pubovesical attachments which suspend the bladder in a high anterior position; in contrast, conventional prostatectomy results in an inferiorly displaced bladder devoid of fixation which experiences increased cephalocaudal compression during Valsalva, leading to stress UI [31]. Retropubic dissection during hernia repair also mobilizes the bladder from the pubis; however, a key difference is the placement of retropubic mesh immediately thereafter [4]. Mesh prostheses induce a foreign body reaction leading to regional fibrosis and complete tissue integration within 2–3 weeks [32]. It is possible the loss of urinary function secondary to pubovesical attachment disruption seen in prostatectomy is avoided after TAR due to rapid de novo pubovesical adhesion formation and restoration of anterior bladder wall tethering to the pubis due to the introduction of a prosthetic material— this could theoretically improve continence for those with intrinsic sphincter or pelvic floor insufficiency by reducing bladder mobility. This is supported by our observation of lower stress incontinence scores in all postoperative cohorts vs the preoperative cohort, a finding that was also observed in the female-only analysis (Early-Post *P* < 0.001; 1-Year *P* = 0.008) and in the matched cohort (*P* = 0.044). Given evidence that mesh weight is positively correlated with inflammatory response, we performed a limited analysis of HWPP vs MWPP which demonstrated a statistically significant decrease in stress UI scores for HWPP recipients vs MWPP recipients and near significance regarding lower postoperative UI prevalence in HWPP recipients (P = 0.069); however, such analyses are exploratory at best and merely hypothesis-generating [33]. Evidence in other surgical fields suggests anterior fixation as a viable theoretical mechanism for stress UI treatment, namely, surgical pubic fixation of the anterior bladder has shown limited promise in the treatment of stress UI in urogynecology literature, but any further exploration of this hypothesis is well-outside the scope of this manuscript [34].

Overall, our QA effort was successful in characterizing UI prevalence at preoperative and early postoperative windows for patients undergoing abdominal wall reconstruction with TAR. These results, however, must be carefully interpreted within the context of many limitations. First, the analysis best captures and characterizes preoperative and early postoperative cohorts— long-term follow-up data are lacking. Though the 1-Year (n = 23) and Distant (n = 7) cohorts did not demonstrate concerning signals regarding UI, they are severely underpowered and may represent an unrepresentative population. This study does not adequately address long-term implications of Retzius space dissection and the absence of observed adverse outcomes within this study does not exclude their possibility. As a QA effort aiming to assess a single-center’s patient population at multiple timepoints, the study’s design favored characterization of UI prevalence at a population level. It was not intended to produce robust, generalizable knowledge, nor was it designed to produce high-quality, prospective data. In a QA/QI fashion, only deidentified data were collected, which limited our ability to audit the dataset for errors and severely limited the number of variables that could be recorded for any given patient. Due to limited patient-specific information capture and small sample sizes, the influence of sampling bias without an ability to further detect or adjust for confounders is a legitimate concern. The matched analysis partially accounted for uncaptured patient factors by theoretically controlling comorbidities and anatomical properties (i.e. body mass index) but was limited by sample size to an even greater extent. Further, though we interpreted our results to infer that space of Retzius dissection during TAR is not associated with UI, due to the deidentified nature of our project there was no reliable method available to confirm that Pre-Op survey participants ultimately underwent space of Retzius dissection unless they were captured in the matched post-operative cohort— though all were consented for the operation. This is discrepant from the post-operative cohorts in whom Retzius was confirmed prior to inclusion. While retropubic dissection is a standard part of TAR at our center, the preoperative group may include participants that did not undergo hernia repair and/or in whom retropubic dissection was limited or avoided and compromises the comparability of pre- and post-operative cohorts.

Our characterization of UI imparts its own, separate set of methodological limitations. The implemented UI definition (UDI-6 score > 33.33) was selected for excellent sensitivity (97%) and specificity (84%) in differentiating symptomatic vs asymptomatic disease in a female population with an age distribution similar to ours, though lower thresholds (namely, > 25) are also employed but with lower specificity [15, 35]. However, inferring UI-related QoL based on UDI-6 score deserves scrutiny. We selected the UDI-6 due to ease of administration and broad capture of multiple aspects of UI. However, the UDI-6 also captures non-UI symptoms— some of which (i.e. lower abdominal pain) may overlap with symptoms of large ventral hernias and/or their repair— and the survey is less useful for tracking UI-specific disease severity in comparison to alternative questionnaires [36, 37]. Further, the UDI-6 can suggest particular UI types, but cannot outright diagnose a sub-type of UI. While some UDI-6 questions correspond to UI type (and we demonstrated apparent trends regarding irritative and stress UI), specificity is low and such associations are not validated [14, 36]. Our findings appear to suggest that the lower UDI-6 scores observed for Early-Post vs Pre-Op cohorts in every comparison (unmatched, matched, female-only, and pathology-free) are clinically meaningful with score differences of 11.1 or greater which is above the most commonly implemented minimal clinically important difference (MCID) of 11 for UDI-6 [38, 39]. However, this should be interpreted with caution— first, multiple MCIDs have been identified for the UDI-6, some of these are based on conversions from the UDI long-form, and variation in clinically important changes has been noted to be both pathology and population-dependent [40–42]. Second, this study was designed to characterize UI burden within outpatient cohorts before or after TAR with Retzius dissection in order to determine if concerns regarding urinary function after TAR were warranted and prospective investigation was warranted, it was not designed as an investigation of treatment effect. Applying an individual-level MCID threshold to population medians is methodologically inappropriate and only prospective investigation designed to assess a treatment effect should be used to draw such conclusions. Overall, the survey was appropriate for the intended purpose of identifying prevalence of symptomatic UI, but the statistical difference observed between total UDI-6 scores (or sub-domain scores) in pre- and postoperative cohorts cannot be deemed clinically significant or meaningful given known limitations of the instrument. Finally, from a practical standpoint, multiple postoperative factors could lead to patient perceptions of decreased UI or transiently decreased burden of UI, particularly stress UI, which may not be reflective of steady-state urinary function. The most common type of UI in the United States is stress incontinence, wherein shifts in abdominal pressure overcome pelvic floor weakness [43]. Abdominal wall reconstruction with TAR is painful and while early mobilization is encouraged, patients self-limit activity to minimize movement of the abdominal wall— avoiding sudden abdominal pressure shifts (like sneezing or quick movements) to prevent pain. Activity avoidance could decrease the quantity of incontinence events and patients may perceive this as an improvement (which may not remain after regular activity levels are resumed). Opioid consumption following abdominal wall reconstruction is another factor of consideration. Opioid-induced bladder dysfunction is a well-recognized clinical entity and opioids are known to increase bladder capacity and suppress detrusor contractility when administered through enteral and parenteral routes, alike [44–47]. Further, limited data from animal studies suggest a therapeutic role of partial mu-agonist opioids (i.e. tramadol) to treat incontinence [48, 49]. Opioid administration, consumption, and post-operative prescribing were not accounted for in this project. Our standard discharge prescription is based on patient utilization within 24 h of discharge; anecdotally, this ranges from 25–75 mg of oxycodone and patients are no longer taking opioids by the time of their follow-up visit (when survey responses were collected), but any opioid-related urinary function improvement may still influence survey responses within the first 30 days. Other aspects of the postoperative course could similarly contribute, acute renal injury is a known risk of abdominal wall reconstruction and decreased urine production may also lead patients to experience fewer incontinence events [50, 51]. These factors, particularly activity restrictions limiting Valsalva and postoperative oliguria reducing urine output, represent plausible mechanisms by which early postoperative physiology could systematically bias UDI-6 scores toward apparent improvement in the absence of any true change in urinary function and cannot be disregarded as potential confounders to the observed findings. The sensitivity analyses which excluded patients still within 3 and 4 weeks demonstrated no difference in results across the early postoperative period, nor did the sub-window analyses which showed persistence of lower UDI-6 scores and stress UI scores among the 77 patients in the 14–30 day window, as well as the 31 patients in the 31–60 day window and the 12 patients in the 61–90 day window.

## Conclusion

This single-center QA initiative identified a 38% prevalence of UI among TAR candidates at our center and found no evidence of increased UI following TARs requiring retropubic dissection. These results most adequately characterize preoperative and early postoperative populations only and offer limited insights into urinary function at extended intervals. Therefore, though preliminarily reassuring, extended follow-up is necessary to better understand long-term implications of Retzius space dissection during abdominal wall reconstruction.

## Supplementary Information

Below is the link to the electronic supplementary material.

Supplementary file1 (DOCX 36 KB)
