# Supplementary material for: Urinary implications of retropubic dissection during abdominal wall reconstruction: Early results from a clinical quality assurance initiative
Source: Hernia. 2026 Jul 17;30(1):293. doi: 10.1007/s10029-026-03771-y (PMC13379416; doi:10.1007/s10029-026-03771-y)
Supplement: Supplementary file 1 — Supplementary file1 (DOCX 36 KB) [file 10029_2026_3771_MOESM1_ESM.docx]

Supplemental Document

1. Supplemental Results: Comparison vs Historical Controls

2. Supplement eTable 1. Comparison of Demographics and Pelvic Pathology in Matched vs Unmatched Patients

3. Supplemental eTable 2. Additional Catheterization, Mesh, and Hernia Characterization of Post-Op Patients

4. Supplemental eTable 3. Sensitivity Analyses Regarding Foley Catheterization

5. Supplemental eTable 4. Analyses of Baseline Pelvic Pathology and Measures of UI

6. Supplement eTable 5. Analysis of Mesh Characteristics and Urinary Outcomes

7. Supplement eTable 6. Sensitivity Analysis of Urinary Outcome Time-Dependency in the Early-Post Cohort

Supplemental Results: Comparison of Study Population vs Historical Cohorts

A total of 235 eligible cases were identified for the concurrent control population (1/1/2025-11/15/2025). Median age was 62 years (IQR 53-69), mean age was 61 years with a standard deviation of 13 and this was non-different vs the overall study, Pre-Op, Early-Post, 1-Year, or Distant cohorts (P>.5 for all). Females comprised 58.5% (n=135), the proportion of which was not different than any study cohort (P>.6 for all).

A total of 555 eligible cases were identified for the historical control population (1/1/2023-12/31/2024). Median age was 61 years (IQR 52-69), mean age was 61 years with a standard deviation of 13, and the population was non-different vs the overall study, Pre-Op, Early-Post, 1-Year, or Distant cohorts (P>.5 for all). Females comprised 59.2% (n=325), the proportion of which was not different than any study cohort (P>.5 for all).

Supplement eTable 1. Comparison of Demographics and Pelvic Pathology in Matched vs Unmatched Patients

| \| 1A. Matched vs Unmatched Participants, Pre-Op \| \| \| \| \| --- \| --- \| --- \| --- \| \| **Variable** \| **Matched Pre-Op** \| **Unmatched Pre-Op** \| ***P*** \| \| n \| 33 \| 105 \|  \| \| Age (y), Median (IQR) \| 65.0 (51.0–69.0) \| 59.0 (52.0–69.0) \| 0.791 \| \| Female, n (%) \| 21 (63.6%) \| 63 (60.0%) \| 0.839 \| \| Urinary incontinence, n (%) \| 11 (33.3%) \| 42 (40.0%) \| 0.543 \| \| UDI-6 Score, Median (IQR) \| 22.2 (11.1–38.9) \| 22.2 (5.6–44.4) \| 0.942 \| \| Irritative Score, Median (IQR) \| 33.3 (16.7–50.0) \| 33.3 (0.0–66.7) \| 0.929 \| \| Stress Score, Median (IQR) \| 16.7 (0.0–33.3) \| 16.7 (0.0–33.3) \| 0.973 \| \| Any urologic/pelvic floor pathology, n (%) \| 5 (15.2%) \| 20 (19.0%) \| 0.797 \| \| BPH (males), n (%) \| 3 (25.0%) \| 14 (33.3%) \| 0.732 \| \| Cystocele/POP (females), n (%) \| 1 (4.8%) \| 3 (4.8%) \| 1.000 \| \| 1B. Matched vs Unmatched Participants, Early-Post \| \| \| \| \| **Variable** \| **Matched Early-Post** \| **Unmatched Early-Post** \| ***P*** \| \| n \| 33 \| 100 \|  \| \| Age (y), Median (IQR) \| 65.5 (51.2–69.0) \| 63.0 (55.0–69.0) \| 0.834 \| \| Female, n (%) \| 22 (66.6%) \| 64 (64.6%) \| 0.836 \| \| Urinary incontinence, n (%) \| 6 (18.1%) \| 18 (18.2%) \| 1.000 \| \| UDI-6 Score, Median (IQR) \| 11.1 (0.0–27.8) \| 11.1 (5.6–27.8) \| 0.678 \| \| Irritative Score, Median (IQR) \| 16.7 (0.0–50.0) \| 16.7 (0.0–41.7) \| 0.928 \| \| Stress Score, Median (IQR) \| 0.0 (0.0–29.2) \| 0.0 (0.0–16.7) \| 0.246 \| \| Any urologic/pelvic floor pathology, n (%) \| 3 (9.1%) \| 21 (21.2%) \| 0.126 \| \| BPH (males), n (%) \| 2 (16.7%) \| 16 (45.7%) \| 0.095 \| \| Cystocele/POP (females), n (%) \| 1 (4.5%) \| 4 (6.2%) \| 1.000 \| |  |
| --- | --- | --- | --- | --- | --- | --- | --- | --- | --- | --- | --- | --- | --- | --- | --- | --- | --- | --- | --- | --- | --- | --- | --- | --- | --- | --- | --- | --- | --- | --- | --- | --- | --- | --- | --- | --- | --- | --- | --- | --- | --- | --- | --- | --- | --- | --- | --- | --- | --- | --- | --- | --- | --- | --- | --- | --- | --- | --- | --- | --- | --- | --- | --- | --- | --- | --- | --- | --- | --- | --- | --- | --- | --- | --- | --- | --- | --- | --- | --- | --- | --- | --- | --- | --- | --- | --- | --- | --- | --- | --- | --- | --- | --- | --- | --- | --- | --- |

Abbreviations: IQR, interquartile range; UDI-6, urinary distress inventory short form; BPH, benign prostatic hyperplasia; POP, pelvic organ prolapse; TAR, transversus abdominis release

Supplemental eTable 2. Additional Catheterization, Mesh, and Hernia Characterization of Post-Op Patients

| **Variable** | **All Postop** | **Early-Post** | **1-Year** | **Distant** |
| --- | --- | --- | --- | --- |
| Foley Catheter Characteristics | |  |  |  |
| n with foley data | 163 | 133 | 23 | 7 |
| Initial foley time (h), Median (IQR) | 25.2 (22.5–42.8) | 25.2 (22.4–34.3) | 24.0 (22.6–36.9) | 46.0 (41.8–68.8) |
| Total foley time (h), Median (IQR) | 25.4 (22.6–45.8) | 25.3 (22.5–43.0) | 25.2 (22.7–50.2) | 46.0 (41.8–68.8) |
| Off ERAS pathway, n (%) | 33 (20.2%) | 24 (18.0%) | 6 (26.1%) | 3 (42.9%) |
| Reason: I/O monitoring, n (%) | 27 (16.6%) | 20 (15.0%) | 5 (21.7%) | 2 (28.6%) |
| Reason: ICU management, n (%) | 13 (8.0%) | 9 (6.8%) | 4 (17.4%) | 0 (0.0%) |
| Reason: Urinary retention, n (%) | 3 (1.8%) | 2 (1.5%) | 1 (4.3%) | 0 (0.0%) |
| Reason: Epidural, n (%) | 1 (0.6%) | 0 (0.0%) | 0 (0.0%) | 1 (14.3%) |
| Second foley placed, n (%) | 11 (6.7%) | 8 (6.0%) | 3 (13.0%) | 0 (0.0%) |
| Second foley time (h), Median (IQR) [if placed] | 26.5 (19.6–34.9) | 21.9 (17.0–32.1) | 32.9 (31.9–34.9) | — |
| Mesh and Hernia Characteristics | |  |  |  |
| n with mesh data | 163 |  |  |  |
| Mesh type distribution |  |  |  |  |
| PP permanent synthetic, n (%) | 151 (92.6%) |  |  |  |
| Heavyweight, n (% of PP) | 101 (66.9%) |  |  |  |
| Mediumweight, n (% of PP) | 50 (33.1%) |  |  |  |
| PTFE permanent biosynthetic, n (%) | 6 (3.7%) |  |  |  |
| Absorbable PLLA/TMC, n (%) | 4 (2.5%) |  |  |  |
| Polyester permanent synthetic, n (%) | 2 (1.2%) |  |  |  |
| Mesh width (cm), Median (IQR) | 30.0 (30.0–40.0) |  |  |  |
| Mesh length (cm), Median (IQR) | 30.0 (30.0–40.0) |  |  |  |
| Mesh area (cm²), Median (IQR) | 900.0 (900.0–1600.0) |  |  |  |
| Hernia width (cm), Median (IQR) | 15.0 (11.0–19.0) |  |  |  |
| Hernia length (cm), Median (IQR) | 22.0 (19.0–26.0) |  |  |  |
| Hernia area (cm²), Median (IQR) | 322.0 (210.0–432.0) |  |  |  |
| Mesh-to-hernia area ratio, Median (IQR) | 3.6 (2.6–4.8) |  |  |  |
| Hernia with flank component, n (%) | 6 (3.7%) |  |  |  |
| Parastomal hernia, n (%) | 9 (5.5%) |  |  |  |

Abbreviations: IQR, interquartile range; ERAS, early recovery after surgery; PP, polypropylene; PTFE, polytetrafluoroethylene; PLLA/TMC, Poly(L-lactide-co-trimethylene carbonate)

Supplemental eTable 3. Sensitivity Analyses Regarding Foley Catheterization

| 3A. Early-Post: Standard-ERAS Pathway vs Off Pathway | | | |
| --- | --- | --- | --- |
| **Variable** | **Standard-ERAS Pathway** | **Off Pathway** | ***P*** |
| n | 109 | 24 |  |
| UI, n (%) | 19 (17.4%) | 5 (20.8%) | 0.770 |
| UDI-6 Score, Median (IQR) | 11.1 (0.0–27.8) | 13.9 (5.6–27.8) | 0.556 |
| Irritative Score, Median (IQR) | 16.7 (0.0–50.0) | 25.0 (0.0–54.2) | 0.418 |
| Stress Score, Median (IQR) | 0.0 (0.0–16.7) | 0.0 (0.0–33.3) | 0.557 |
| 3B. Early-Post: No second foley vs Second foley placed | | | |
| **Variable** | **Standard-ERAS Pathway** | **Off Pathway** | ***P*** |
| n | 125 | 8 |  |
| UI, n (%) | 22 (17.6%) | 2 (25.0%) | 0.635 |
| UDI-6 Score, Median (IQR) | 11.1 (0.0–27.8) | 19.4 (5.6–37.5) | 0.328 |
| 3C. Matched-Cohort Foley Time (hours) vs ΔUDI-6 | | | |
| **Analysis** | **n** | **Spearman rho** | ***P*** |
| Foley Time vs ΔUDI-6 (matched cohort) | 33 | -0.181 | 0.312 |
| Foley Time vs ΔIrritative Score (matched cohort) | 33 | -0.138 | 0.444 |
| Foley Time vs ΔStress Score (matched cohort) | 33 | -0.158 | 0.381 |

Abbreviations: ERAS, early recovery after surgery; UI, urinary incontinence; UDI-6, urinary distress inventory short form; IQR, interquartile range.

Supplemental eTable 4. Analyses of Baseline Pelvic Pathology and Measures of UI

| 4A. Association Analysis of Urologic or Pelvic Pathology and UI Prevalence and UDI-6 Among Pre-Op Patients | | | |
| --- | --- | --- | --- |
| **Variable** | **Pathology Present** | **No Pathology** | **P** |
| Pre-Op: Any pathology vs No pathology | n=25 | n=113 |  |
| Urinary incontinence, n (%) | 11 (44.0%) | 42 (37.2%) | 0.650 |
| UDI-6 Score, Median (IQR) | 27.8 (16.7–38.9) | 22.2 (5.6–44.4) | 0.245 |
| Pre-Op Females: Cystocele/POP vs None | n=4 | n=80 |  |
| Urinary incontinence, n (%) | 1 (25.0%) | 34 (42.5%) | 0.637 |
| UDI-6 Score, Median (IQR) | 25.0 (20.8–30.6) | 27.8 (11.1–44.4) | 0.966 |
| Pre-Op Males | n=17 | n=37 |  |
| Urinary incontinence, n (%) | 7 (41.2%) | 11 (29.7%) | 0.536 |
| UDI-6 Score, Median (IQR) | 27.8 (11.1–44.4) | 11.1 (0.0–33.3) | 0.055 |
| 4B. Sensitivity Analysis of Primary Analyses Restricted to Patients without Pathology, Pre-Op vs Early-Post Cohorts | | | |
| **Variable** | **Pre-Op without Pathology** | **Early-Post without Pathology** | **P** |
| n | 113 | 109 |  |
| Age (y), Median (IQR) | 59.0 (51.0–68.0) | 62.0 (54.0–69.0) | 0.138 |
| Female, n (%) | 77 (68.1%) | 81 (74.3%) | 0.374 |
| Time since TAR (d), Median (IQR) | — | 25 (17–33) | N/A |
| Urinary incontinence, n (%) | 42 (37.2%) | 20 (18.3%) | 0.003 |
| UDI-6 Score, Median (IQR) | 22.2 (5.6–44.4) | 11.1 (0.0–27.8) | 0.001 |

Abbreviations: UDI-6, urinary distress inventory shortform; IQR, interquartile range; POP, pelvic organ prolapse; TAR, transversus abdominis release

Supplement eTable 5. Analysis of Mesh Characteristics and Urinary Outcomes

| 5A. Heavy-weight vs Medium-Weight Polypropylene and Urinary Outcomes Among Early-Post Patients | | | |
| --- | --- | --- | --- |
| **Variable** | **HWPP** | **MWPP** | ***P*** |
| n | n=86 | n=37 |  |
| UI, n (%) | 11 (12.8%) | 10 (27.0%) | 0.069 |
| UDI-6 Score, Median (IQR) | 11.1 (0.0–26.4) | 16.7 (5.6–33.3) | 0.139 |
| Irritative Score, Median (IQR) | 16.7 (0.0–33.3) | 33.3 (0.0–33.3) | 0.145 |
| Stress Score, Median (IQR) | 0.0 (0.0–16.7) | 0.0 (0.0–33.3) | 0.024 |
| Mesh area (cm²), Median (IQR) | 900.0 (900.0–900.0) | 1600.0 (1200.0–2500.0) | <0.001 |
| Hernia area (cm²), Median (IQR) | 286.0 (190.0–375.0) | 390.0 (252.0–532.0) | 0.003 |
| Mesh-to-hernia ratio, Median (IQR) | 3.1 (2.4–4.5) | 4.1 (3.5–5.4) | 0.006 |
| 5B. Association Analysis of Mesh Size with Change in UDI-6 Among Matched Patients | | | |
| **Analysis** | **n** | **Spearman rho** | ***P*** |
| Mesh area vs ΔUDI-6 (matched, all mesh types) | 33 | -0.119 | 0.511 |
| Hernia area vs ΔUDI-6 (matched, all mesh types) | 33 | -0.194 | 0.280 |
| Mesh:hernia ratio vs ΔUDI-6 (matched, all mesh types) | 33 | 0.169 | 0.347 |
| Mesh area vs ΔIrritative Score (matched, all mesh types) | 33 | -0.015 | 0.934 |
| Hernia area vs ΔIrritative Score (matched, all mesh types) | 33 | -0.015 | 0.935 |
| Mesh area vs ΔStress Score (matched, all mesh types) | 33 | -0.222 | 0.215 |
| Hernia area vs ΔStress Score (matched, all mesh types) | 33 | -0.361 | 0.039 |
| Mesh:hernia ratio vs ΔStress Score (matched, all mesh types) | 33 | 0.272 | 0.126 |

Abbreviations: HWPP, heavy-weight polypropylene; HWPP, medium-weight polypropylene; UI, urinary incontinence; UDI-6, urinary distress inventory shortform; IQR, interquartile range.

Supplement eTable 6. Sensitivity Analyses of Urinary Outcome Time-Dependency in the Early-Post Cohort

| 6A. Association Analysis of Post-Operative Days and UDI-6 Score Among Pre-Specified Cohorts | | | |
| --- | --- | --- | --- |
| **Analysis** | **n** | **Spearman rho** | ***P*** |
| Days post-TAR vs UDI-6 Score — Early-Post (all) | 133 | 0.081 | 0.353 |
| Days post-TAR vs UDI-6 Score — Early-Post (females only) | 86 | 0.055 | 0.617 |
| Days post-TAR vs Irritative Score — Early-Post (all) | 133 | 0.061 | 0.485 |
| Days post-TAR vs Stress Score — Early-Post (all) | 133 | 0.073 | 0.406 |
| Days post-TAR vs UI (binary) — Early-Post (all) | 133 | 0.026 | 0.767 |
| Days post-TAR vs ΔUDI-6 — Matched cohort | 33 | -0.071 | 0.694 |
| Days post-TAR vs ΔIrritative Score — Matched cohort | 33 | -0.167 | 0.354 |
| Days post-TAR vs ΔStress Score — Matched cohort | 33 | 0.03 | 0.871 |

| 6B. Sensitivity Analysis of Early-Post Urinary Outcomes if Surveys from <21 and <30 days are Excluded | | | | | | | |
| --- | --- | --- | --- | --- | --- | --- | --- |
| **Variable** | **Pre-Op** | **Early-Post, >21d** | ***P*^a^** | **Early-Post, >30d** | ***P*^a^** | |  |
| n | 138 | 88 |  | 43 |  |  |  |
| Urinary incontinence, n (%) | 53 (38.4%) | 15 (17.0%) | <0.001 | 5 (11.6%) | <0.001 |  |  |
| UDI-6 Score, Median (IQR) | 22.2 (11.1–44.4) | 11.1 (5.6–27.8) | 0.001 | 11.1 (0.0–25.0) | 0.001 |  |  |
| Irritative Score, Median (IQR) | 33.3 (16.7–50.0) | 16.7 (0.0–33.3) | 0.025 | 16.7 (0.0–33.3) | 0.022 |  |  |
| Stress Score, Median (IQR) | 16.7 (0.0–33.3) | 0.0 (0.0–16.7) | 0.001 | 0.0 (0.0–16.7) | <0.001 |  |  |
| 6C. Comparison of Pre-Op Demographic and Urinary Outcomes vs Sub-Windows of the Early-Post Cohort | | | | | | | |
| **Variable** | **Pre-Op** | **14-30d** | ***P*^a^** | **31-60d** | ***P*^a^** | **61-90d** | ***P*^a^** |
| n | 138 | 77 |  | 31 |  | 12 |  |
| Age (y), Median (IQR) | 60.5 (52.0–69.0) | 63.0 (54.0–71.0) | 0.228 | 63.0 (55.0–68.0) | 0.596 | 64.5 (55.5–67.2) | 0.782 |
| Female, n (%) | 84 (60.9%) | 50 (64.9%) | 0.660 | 22 (71.0%) | 0.314 | 7 (58.3%) | 1.000 |
| Time since TAR (d), Median (IQR) | — | 22 (19–27) | N/A | 35 (32–40) | N/A | 38 (8–80) | N/A |
| Urinary incontinence, n (%) | 53 (38.4%) | 17 (22.1%) | 0.015 | 3 (9.7%) | 0.001 | 2 (16.7%) | 0.212 |
| UDI-6 Score, Median (IQR) | 22.2 (11.1–44.4) | 13.3 (5.6–27.8) | 0.011 | 11.1 (0.0–25.0) | 0.007 | 8.3 (4.2–19.4) | 0.029 |
| Irritative Score, Median (IQR) | 33.3 (16.7–50.0) | 33.3 (0.0–50.0) | 0.162 | 16.7 (0.0–41.7) | 0.083 | 16.7 (0.0–33.3) | 0.073 |
| Stress Score, Median (IQR) | 16.7 (0.0–33.3) | 0.0 (0.0–16.7) | 0.008 | 0.0 (0.0–16.7) | 0.009 | 0.0 (0.0–0.0) | 0.019 |

Abbreviations: IQR, interquartile range; TAR, transversus abdominis release; UDI-6, urinary distress inventory short-form.

Mann-Whitney U test utilized for continuous variable comparisons and Fisher’s Exact test used for categorical variable comparisons.

a. Comparison vs Pre-Op
